# 

**Published:** 1853-07

**Authors:** 


### SELECTED ARTICLES.

Risodontrypy; or, Treatment of Exposed Nerves. By C. O. Cone, M. D. .......... 221
Examination of the Teeth .......... 225

### DENTAL ABSTRACTS.

Deep-seated Dental Caries. By W. H. Dwinelle .......... 229
The April Number of the Dental News-Letter .......... 234
New Styptic .......... 241
Horned Woman .......... 242
Report of the Organization of the Dental Association of Allegheny county, Pa. .......... 243

### EDITORIAL.

American Dental Patents. By Geo. W. Kendall .......... 245
Sources of Vitality of the Teeth .......... 247
Specimens of Artificial Teeth .......... 248
Our Dental Abstracts .......... 248
Present Number of the Register .......... 248
Practical Hints on the Teeth .......... 249
Subscribers .......... 249
Sad Casuality .......... 249
Postage .......... 249
John D. Chevalier .......... 250
Thompson's Compound Self-adjusting Blow-pipe .......... 250
Stockholders of the Ohio College of Dental Surgery, .......... 250
Journals received .......... 250

